# Endoscopic Evaluation for Stricture Formation Post Button Battery Ingestion

**DOI:** 10.3390/pediatric13030059

**Published:** 2021-09-01

**Authors:** Amber Bulna, Amanda C. Fifi

**Affiliations:** Division of Pediatric Gastroenterology, Hepatology & Nutrition, Department of Pediatrics, Miller School of Medicine, University of Miami, Miami, FL 33136, USA; afifi@med.miami.edu

**Keywords:** button battery, esophogastroduodenoscopy, stricture

## Abstract

Every year, there are over 3300 ingestions of button batteries, mostly by young children. Initial presentation of button battery ingestion may be nonspecific, with a delay in diagnosis and removal resulting in increased risk of complications. We present the case of a five-year-old female who presented with vomiting following unwitnessed button battery ingestion. The battery was impacted in the middle esophagus for at least six hours. Endoscopy was performed for immediate removal and showed a Grade 2B erosion, warranting nasogastric tube placement. The patient remained asymptomatic following discharge and had a barium swallow that was read as normal. However, a repeat endoscopy one month later visualized stricture formation at the previous battery injury site. This case highlights the importance of both clinician and parent awareness of button battery ingestion and demonstrates that endoscopy provides the most accurate assessment of esophageal injury and complication development, even in asymptomatic patients.

## 1. Introduction

Every year, there are over 3300 ingestions of button batteries, mostly by young children between six months and three years old. Unfortunately, the incidence is rising due to increased use in household products and compact electronics, with common sources being hearing aids and toys [1,2]. The National Poison Data System shows a 6.7-fold increase in button battery ingestions resulting in devastating outcomes from 1985 to 2009 [3]. Therefore, it is crucial to spread awareness and ensure parents have the proper education for avoidance of ingestions and physicians have clear guidelines for their management.

## 2. Case Presentation

**Figure 1:**
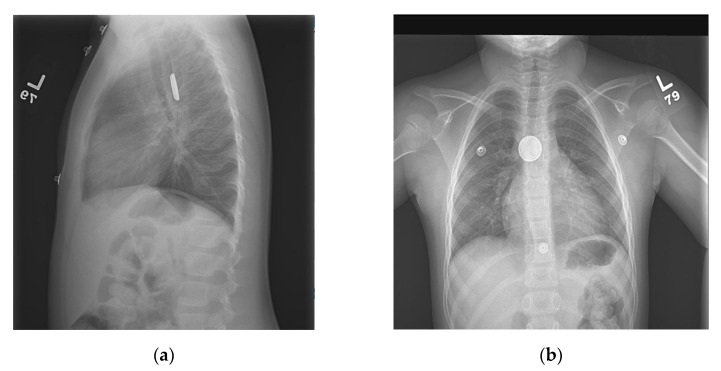
(**a**) Lateral chest X-ray showing the button battery with a step-off appearance in the mid-esophagus; (**b**) AP chest X-ray showing the button battery with a double halo/ring sign in the mid-esophagus.

The patient is a five-year-old female with a past medical history of Wolff–Parkinson– White syndrome, asthma, and oppositional defiant disorder who presented to the emergency department with unwitnessed ingestion of a foreign body. The patient’s mother endorsed that on 29 November 2020 at 21:00, the patient complained of chest pain and admitted to previously swallowing a silver object. Upon arrival at the hospital, she had four episodes of non-bloody, nonbilious emesis, and epigastric pain. A chest X-ray showed a round metallic object projecting over the mid esophagus, with a double halo and step-off appearance (Figure 1), consistent with button battery (BB) ingestion. Due to the potential for rapid onset of damage following BB ingestion, the patient was subsequently admitted for emergent endoscopic removal of the BB, and directions were given that she was to receive nothing by mouth (NPO).

**Figure 2:**
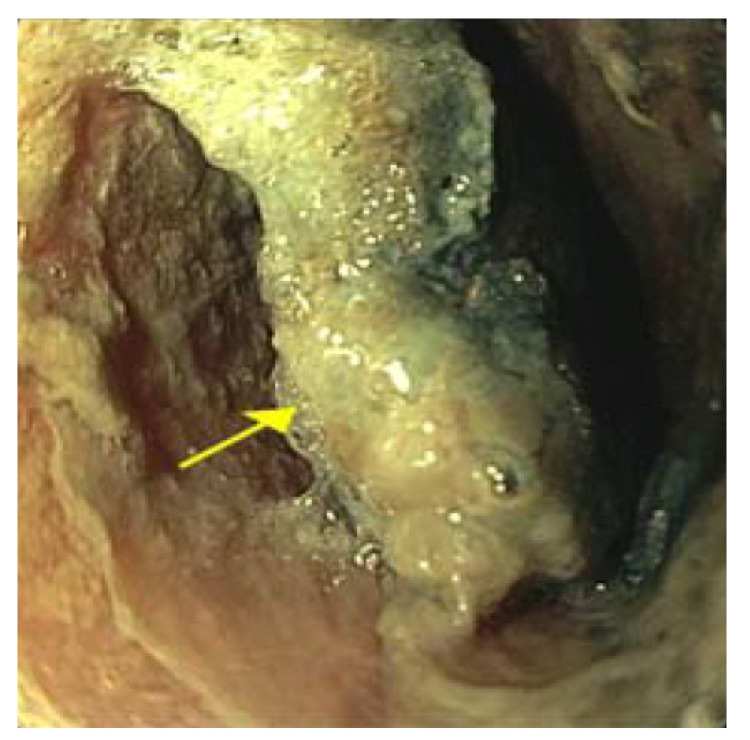
Endoscopy six hours following initial ingestion of the button battery. It shows significant wall necrosis with the yellow arrow identifying the button battery impacted in the middle esophagus and covered with secretions.

**Figure 3:**
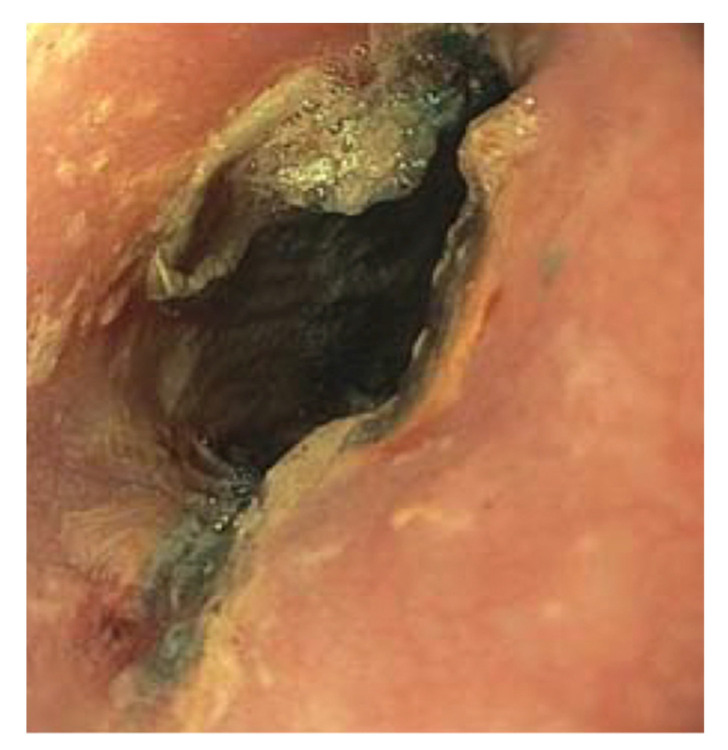
Endoscopy showing wall necrosis of the middle esophagus immediately following button battery removal.

We performed an esophogastroduodenoscopy (EGD) 6 h after ingestion and discovered a 20 mm BB in the middle third of the esophagus (Figure 2). We successfully removed the BB with rat tooth forceps without complications. Visualization of the site showed mucosal ulceration and erosion (Figure 3). According to the commonly used Zargar classification of esophageal injuries, this lesion was determined to be Grade 2B due to the erosion’s circumferential nature. The endoscope was advanced to the stomach and showed no damage. A nasogastric (NG) tube was not placed at the time of the endoscopy.

**Figure 4:**
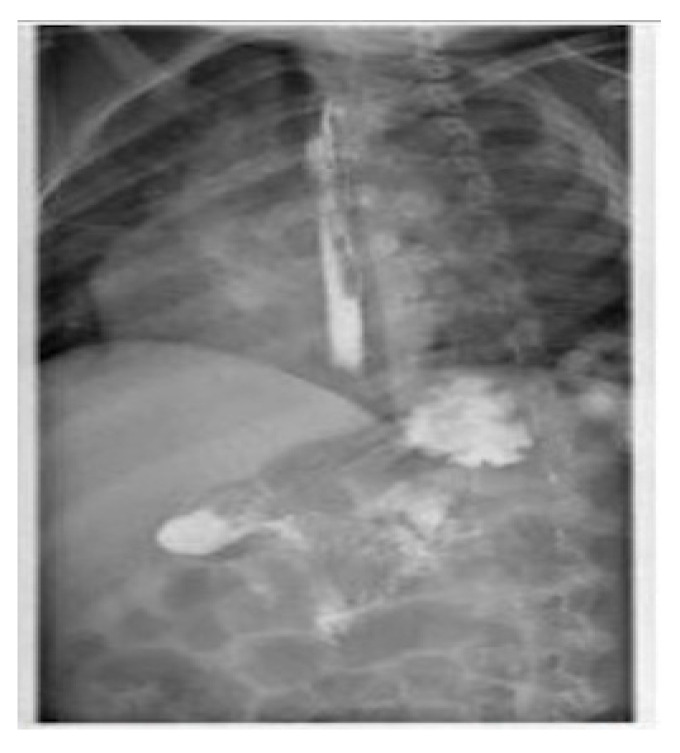
Esophagram demonstrating irregularity in the middle esophagus, with no extravasation of fluid.

The following day, the patient endorsed substernal chest pain and dysphagia with saliva. Her diet remained NPO, and she was started on ceftriaxone and intravenous (IV) pantoprazole. A follow-up chest X-ray on day two showed no evidence of cardiopulmonary disease, pneumomediastinum, or pneumothorax. An esophagram on day three showed mild irregularity in the mid-esophagus but no contrast extravasation to suggest perforation (Figure 4).

**Figure 5:**
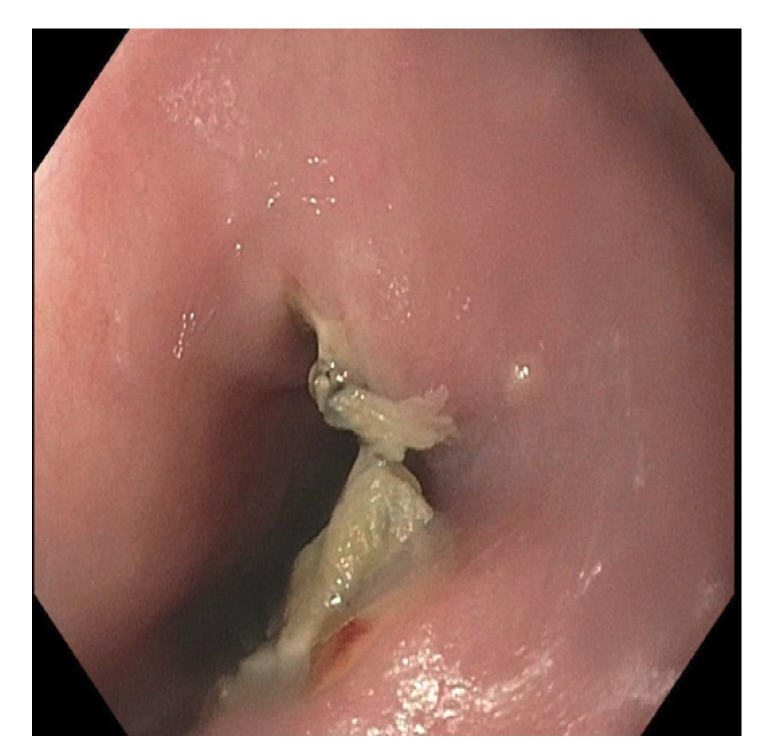
Endoscopy four days following the button battery ingestion. It demonstrates a single, non-bleeding erosion and necrotic tissue in the middle esophagus.

We performed a repeat EGD on day four to assess esophageal mucosa healing and place an NG tube for feeds. The endoscope was progressed to the middle third of the esophagus, revealing a single, non-bleeding, posterior erosion with sloughing of necrotic tissue (Figure 5). A 10 French NG tube was placed under direct visualization. Post endoscopy, we initiated feeds via NG tube, but the patient developed a fever of 38.8 °C. We then added IV cefepime and metronidazole to her medication regimen and held feeds due to the concern for mediastinitis. An MRI showed possible inflammation in the posterior mediastinum. However, the patient’s fever subsided, and a chest X-ray did not reveal signs of esophageal perforation. On day six, we restarted feeds via NG tube and discontinued antibiotics. On day eight, a repeat esophagram showed persistent irregularity of the posterior esophageal wall consistent with ulceration. She was discharged the same day on NG tube feeds, omeprazole, and instructed to avoid food and water by mouth.

The patient returned to the ED the following day with bilious vomiting following bolus tube feeds. An X-ray revealed that the tip of the NG tube had been displaced to the duodenum, which was then pulled back to the stomach without complication.

**Figure 6:**
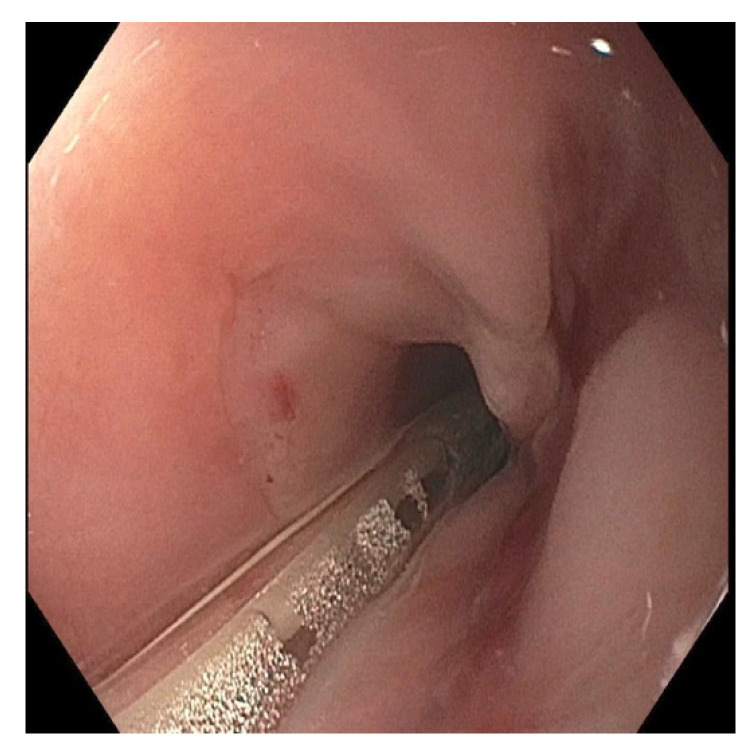
Endoscopy showing esophageal stenosis with friable mucosa in the middle esophagus, one month following button battery ingestion and removal.

**Figure 7:**
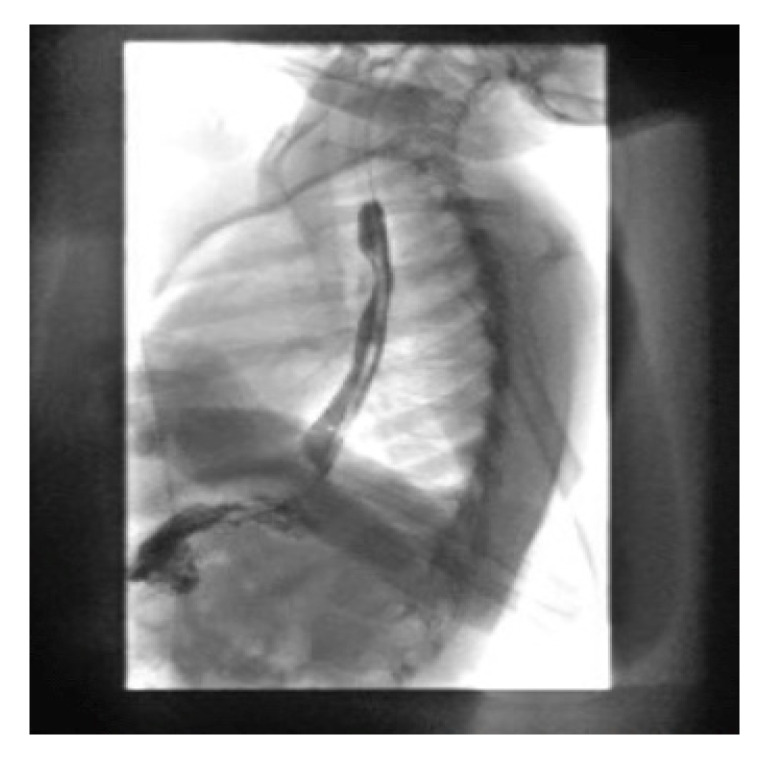
Esophagram approximately one month following initial ingestion of the button battery. There was no evidence of stricture or perforation.

At clinic follow-up 4 weeks following ingestion, the patient was tolerating feeds via NG tube and sips of water. The following day, we used a GIF-HQ 190 endoscope of 9.9 mm diameter that showed moderate stenosis with friable mucosa in the middle third of the esophagus (Figure 6). We could not traverse the stenosis with the endoscope. Due to the lack of equipment available and the endoscopist’s minimal experience with strictures, we did not attempt dilation at that time. We performed an esophagram two days later to assess the length of the stricture, but there was no evidence of strictures, perforation, or fistulas on imaging in neither the lateral view (Figure 7) or the AP view.

**Figure 8:**
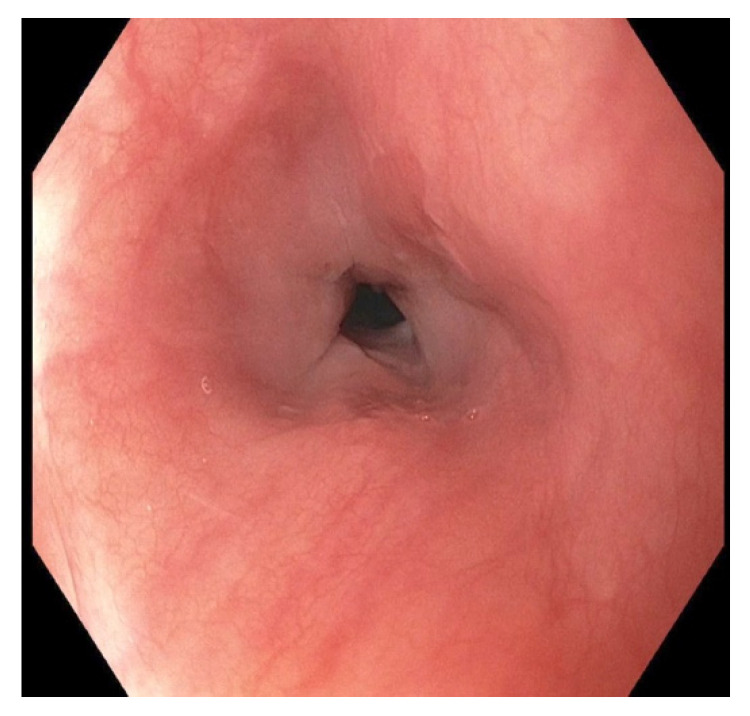
Endoscopy seven weeks following initial ingestion and removal of the button battery, showing esophageal stenosis that could not initially be traversed by the endoscope.

**Figure 9:**
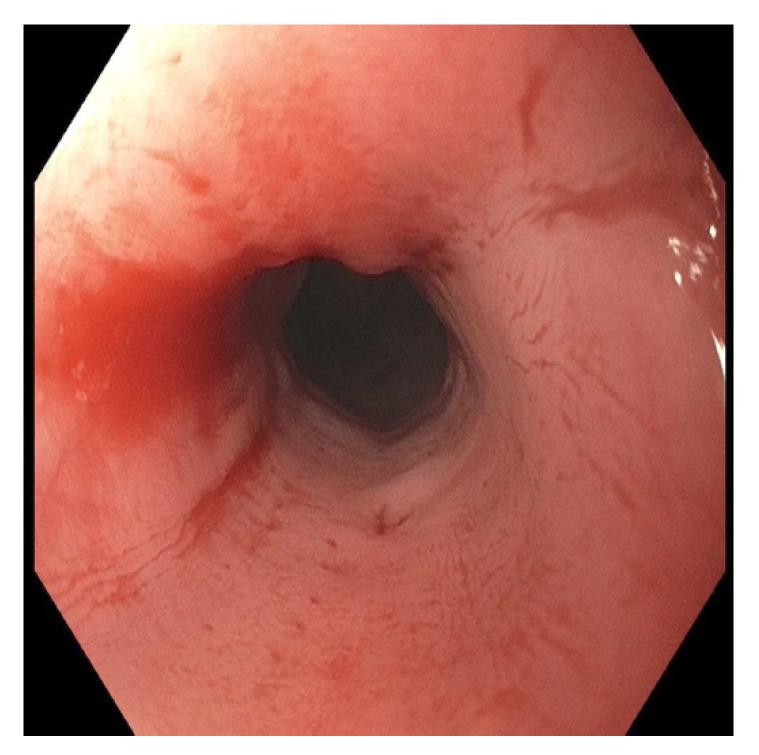
Endoscopy of the middle esophagus following balloon dilation of the stricture seven weeks post button battery ingestion.

About seven weeks post ingestion, she returned for an EGD with possible stricture dilation and NG tube removal. The patient was tolerating a full liquid diet by mouth in addition to NG tube feeds, with no symptoms of fever, chest pain, or dysphagia. Upon introduction, the endoscope could not pass through a severe esophageal stenosis 15 cm from the incisors (Figure 8). Balloon dilation was performed to 10 mm, 12 mm, 13.5 mm, and twice to 15 mm (Figure 9), after which we passed the endoscope to the stomach and removed the NG tube. The patient tolerated the procedure well and was discharged to home once she tolerated liquids by mouth.

## 3. Discussion

Over 97% of cases are either mild or asymptomatic with no significant outcome [4]. However, significant complications have been noted in the literature, with damage not only to the site of impaction but to surrounding structures as well. These include perforations, tracheoesophageal fistulas, aortic rupture, vocal cord paralysis, esophageal strictures, and mediastinitis. It has been shown that 27% of cases with major outcomes and 54% of cases resulting in fatality were initially misdiagnosed [3,4]. In addition, parents often do not notice their children ingesting the battery, with 92% of fatalities and 56% of major outcome cases being unwitnessed [3]. BB ingestion may present vaguely, with common symptoms of fever, cough, vomiting, and dysphagia. These may suggest an alternate diagnosis unless an initial chest X-ray reveals the battery. Physicians should also note the increasing number of foreign body ingestions among patients with psychiatric conditions, such as autism, and those with a history of anemia [5,6]. Fever in combination with dysphagia is more specific for and ingested object located in the esophagus, especially in toddlers. Lateral and anteroposterior X-rays are required to confirm the presence of a BB, distinguishable by its double density and presence of step-off between electrodes [7,8,9]. A BB impaction in the esophagus should be removed immediately. In our case, the patient fortunately admitted to swallowing something. However, the exact object swallowed was in question and she had mild, vague symptoms. Multiple chest X-rays in different views were conducted to make an accurate diagnosis. This demonstrates the importance of initial X-rays and accurate identification of the ingested object, especially due to the rapidity at which damage develops. Necrosis of the esophagus can occur within 15 min of BB ingestion, with extension to the outer esophageal layer within 30 min [10,11]. Our case had a six-hour window between battery ingestion and removal and resulted in significant ulceration of mucosa with eventual stricture formation.

BB should be removed immediately, preferably using an endoscope, which allows for direct visualization of mucosal injury [12]. However, due to the electrical current and hydroxide production, which has been shown to cause more significant harm than actual pressure at the site of impaction, erosion may continue despite removal of the battery [8,13]. Weak acidic solutions have been shown to possibly decrease the rate of esophageal injury [14]. The European Society for Pediatric Gastroenterology Hepatology and Nutrition suggests considering honey or sucralfate if the patient is stable, can swallow, and it has been under 12 h since ingestion. However, this should not delay endoscopy [15].

Meticulous monitoring of the patient is necessary for adequate management of possible complications, both immediately following removal and after the patient is discharged. Necrosis is the most likely sequelae in the first week, followed by ulceration that could potentially result in perforation, and finally formation of strictures secondary to scar formation [16]. Patient presentation may not correlate with degree of damage, so follow-up imaging and endoscopy must be taken into consideration [15]. For example, our patient had only minimal symptoms of a mild burning sensation with water consumption 4 days following BB removal, but an EGD showed moderate sloughing and necrotic tissue at the previous battery site. This highlights the need for repeat endoscopy within the first week to assess for injury progression and guide further management.

Many factors influence decisions in management following endoscopic removal of BB. The location and extent of damage as well as the time elapsed between ingestion and removal must be considered to guide follow-up imaging, reintroduction of feeds, medication administration, and safe discharge. However, post endoscopic care is challenging and varies based on each individual patient [17]. We chose to follow guidelines for Grade 2B erosions that include placing an NG tube and monitoring inpatient for at least 48 h. We also administered antibiotic prophylaxis and a proton pump inhibitor (PPI) to allow for proper healing of the esophageal mucosa. PPIs have been shown to promote mucosal healing and decrease the risk of stricture formation in the setting of corrosive esophageal injury [18,19]. While steroids have been suggested to prevent stricture formation, there is a lack of supporting evidence in the literature for this practice [20]. We therefore decided to withhold steroid treatment.

Patients are normally discharged once they tolerate feeds and show no signs of perforation. However, they still require strict follow up, for complications and fatality may still occur long after discharge. Strictures can form even months following initial presentation [8]. In severe cases, a lateral neck dissection may be the only option for intervention [21]. We knew it was imperative to follow up with our patient to monitor for adverse sequelae. Current recommendations suggest that cases involving significant ulceration warrant an esophagram at four weeks to evaluate for long-term sequelae; if this shows no evidence of complication, observation is no longer necessary in the absence of new symptom onset [22]. While guidelines are available for management immediately following BB removal, they are not definite for long-term follow up. An upper GI series 2–3 weeks following ingestion has been suggested in cases of Grade 2B erosions. However, there is still no agreement on whether an endoscopy should be preferred over a contrast study. In this case, our patient received an esophagram and underwent a repeat EGD, one month following initial presentation. She was asymptomatic and tolerating a full liquid diet by mouth. While the endoscopy visualized moderate stenosis, the esophagram showed no evidence of stricture. EGD allowed for a more accurate assessment of the esophagus and timely intervention in the setting of stricture formation. We therefore conclude that EGD should be used over esophagram when evaluating for long-term complications following BB ingestion with moderate to severe ulceration, even in the absence of symptoms suggesting stricture formation.

Manufacturers should be aware of the dangers button batteries pose to children. Due to worse outcomes associated with larger batteries, small ones should be used when possible [4]. In addition, button batteries should be tightly secured, requiring a screw driving or twisting mechanism to be removed [13]. Physicians must also remain actively aware of BB ingestion in the pediatric population and able to make accurate diagnoses. BB ingestions should be emphasized in early medical curriculums and pediatric training programs. While there are recommendations in place for immediate removal and diagnostic imaging, physicians need clear guidelines to direct management in both the short- and long-term time periods following initial BB esophageal injury [15].

## 4. Conclusions

BB ingestion may result in devastating complications, especially with delays in management. It is therefore essential that clinicians include this on their differentials when children present with symptoms suggestive of ingestion without delay. Careful evaluation of chest X-rays allows for timely intervention. Endoscopy provides more accurate assessment of esophageal injury and long-term complications than an esophagram. We must increase awareness of the dangers surrounding button batteries in the pediatric population. While parents should practice caution with BB use in the home, health professionals need adequate education and guidelines in place to effectively manage immediate and long-term complications in children presenting with BB ingestion.

## Data Availability

Not applicable.
